# Valuing home modifications: The street‐level policy work of occupational therapists in Australian home modification practice

**DOI:** 10.1111/1440-1630.12836

**Published:** 2022-08-22

**Authors:** Grace Bitner, Coral Gillett, Michele Foster

**Affiliations:** ^1^ The Hopkins Centre, Menzies Health Institute Queensland Griffith University Nathan Queensland Australia

**Keywords:** ageing, disability, health, home modification, occupational therapy, value, value for money

## Abstract

**Introduction:**

Occupational therapists recommending home modifications in Australia are often required by funding bodies to consider ‘value’ and ‘value for money’ (VFM); however, clear guidance on how to define and apply these concepts is not always provided. This paper reports on a qualitative study examining how the concepts of value and VFM are currently understood and operationalised by occupational therapists in Australian home modification practice, with the aim of positively contributing to both policy and practice in this area.

**Methods:**

The study utilised constructivist grounded theory to collect and analyse qualitative data from 20 occupational therapists who were currently working across Australia and had professional experience in home modifications.

**Findings:**

The grounded theory that was derived from the analysis highlights the unique position that occupational therapists occupy in home modification work as they strive to align the values of different stakeholders to create solutions that all consider to be valuable. In the absence of consistent frameworks or methods for determining value and VFM, evidence also emerged of occupational therapists using a range of individual approaches such as using formal and informal care as metrics, cheapest option approaches, and comparative costing.

**Conclusion:**

In addition to a clear need for consistent and transparent approaches to understanding and operationalising VFM in home modifications, there is also a need for further investigation into the value systems that underpin this work. A conceptualisation of occupational therapists as street‐level policy agents has proven useful here as it highlights the position occupational therapists occupy, enacting, making, and, at times, challenging policy in day‐to‐day practice as they work to align the values of the various stakeholders.

**Key Points for Occupational Therapy**

- Value‐for‐money (VFM) determinations in home modification work are complex and multidimensional.
- Occupational therapists regularly engage in ‘value alignment’ work between stakeholders to create solutions that are considered to represent VFM.
- Clear and consistent approaches to determining VFM in home modifications are needed.

## 1 INTRODUCTION AND CONTEXT

This paper reports on the findings of a qualitative study into the concepts of value and value for money (VFM) in Australian home modification practice. In Australia, home modifications for individuals with disability (of all ages) are currently funded by a wide range of organisations and bodies, from state and federal government programmes through to private and public insurance schemes. The introduction of the National Disability Insurance Scheme (NDIS) further expanded this sector, providing more individuals access to funds for home modifications (Disability Housing Futures Working Group, 2016). Within these schemes, the concepts of value and VFM are often central to funders' decision making as to which modifications will be approved. For the occupational therapists tasked with recommending the home modifications, they can be required to make decisions surrounding, and provide evidence and estimations of, value and VFM to funders as part of their work. Despite this requirement, clear guidance as to how these concepts are to be defined, applied, and determined is not always readily available. There also can be significant difference between the many funding bodies as to what is considered valuable and constitutes VFM.

In the context of occupational therapy practice, de Jonge and Hoyle's (2019) definition of value in occupational therapy as ‘… the importance or significance of the occupation to the individual’ (p. 124) is useful here. It does not necessarily conceptualise value as being fixed or discrete—but rather relative—allowing for individual's values to also change over time (de Jonge & Hoyle, 2019, p. 124). Aplin et al. (2013) describe the intertwined nature of occupation with the physical environment, making the home a ‘rich and meaningful environment’ (p. 102), and identify home modifications as ‘key to enhancing this valued dimension of home’ (p. 103) for older adults and people with a disability. However, Martel (2017) has pointed out that:

Value is not something that is easy to grasp when you look closely at it. Value as a noun describes the regard in which something is held, its importance, worth, or usefulness. As a verb, valuing is the act of estimating the monetary worth of that thing. Getting the balance right when attempting to monetise the usefulness of something is almost always a non‐precise judgement call. (p. 19)

VFM is similarly a complex term. The field of Health Economics provides evaluation tools such as cost–benefit analysis to quantify VFM, and some have adapted these tools to be used in the evaluation of home modification and occupational therapy interventions more broadly (Bejerholm et al., 2020; Green & Lambert, 2017; Heywood & Turner, 2007; Lambert et al., 2014). At a societal level, VFM is often assessed according to alternative frameworks such as social return on investment (Banke‐Thomas et al., 2015). Smith (2009) broadly defines VFM in health care as being about ‘how resources are successfully transformed into valued outcomes’(p. 12). In relation to home modifications specifically, Heywood (2001) describes VFM as ensuring that ‘any adaptation carried out is truly the most beneficial option from the viewpoint of the family or individual, and the most sensible use of money.’ (p. 37).

Although the terms home modification and home adaptation are often used ‘… interchangeably …’ (Chiatti & Iwarsson, 2014, p. 324), this study employs the term home modification. As used here, home modification includes not only modifications to the built form of a dwelling (ranging from simple to complex) but also assistive or adaptive technology, as many Australian funding schemes group these together. This broad definition adopted was supported by participant's consistent references to assistive technology as part of their home modification practice.

Within the home modification literature, there is growing interest in how the matters of resource distribution and VFM are dealt with in practice. In particular, increasing attention is being paid to both health economics and the ethical facets of occupational therapists' decision making in home modification practice (Chiatti & Iwarsson, 2014; Dige, 2009; Johansson et al., 2010; Kinsella et al., 2008; Sandman, 2012; Vanderkaay et al., 2019, 2020). In 2014, Chiatti and Iwarsson highlighted the potential that a range of economic decision‐making tools, such as cost–benefit analysis, cost‐effectiveness analysis, and cost‐utility analysis, held for occupational therapists undertaking home modification work. Since then, others have examined the intersection between home modification and health economics from a range of vantage points, including the cost–benefits of preventative home modification interventions (Garrett et al., 2016), evaluations of cost‐effectiveness (Powell et al., 2017; Roys et al., 2016; Zhang & Zhou, 2020), the potential for home modifications to save costs in a range of other public health domains (Garrett et al., 2016; Garrett & Roys, 2017), determinations around the potential ‘health gain, cost‐utility and health equity impacts …’ (Pega et al., 2016), and return on investment (Garrett & Roys, 2017; Powell et al., 2017).

Interwoven in these discussions of resource distribution and VFM, however, is recognition of the need to also better understand the values that underpin and guide this decision making. Johansson (2013) has underscored the need to better understand ‘… how values are enacted …’ (p. 414) in home modification practice, highlighting that ‘decision‐making in health care cannot be understood without including how values are acted and experienced in encounters between patients, clients, and different groups of professionals.’ (p. 426). Similarly, a number of researchers have explored how occupational therapists navigate the ‘ethical tensions’ related to balancing client priorities, and values, with those of other stakeholders such as funding bodies and the structures and systems within which they work (Bushby et al., 2015; Durocher et al., 2016; Kinsella et al., 2008). Others have drawn attention to the importance of occupational therapists' personal values, highlighting the need to ‘… understand how such internalized values come into play within everyday practice‐based decision making …’ (Clair & Newcombe, 2014, p. 155). Vanderkaay et al. (2020) identify the need for further research to ‘… elucidate the relationship of personal values and ethical decision‐making’ (p. 109) in occupational therapy practice. Also notably missing from the literature is research investigating how the concepts of value and VFM are currently understood and operationalised at the grassroots level by practitioners. This question is particularly pertinent to the Australian context where—despite updates to the guidance provided surrounding VFM determinations—both the Ernst and Young (2015) and subsequent Tune (2019, p. 220) independent reviews of the NDIS Act 2013 concluded that there was an ongoing need to ‘amend the Supports for Participants Rules to provide further guidance on how value for money could be determined’. This was part of a broad set of recommendations that collectively sought to provide Australians with disabilities ‘more certainty on the role of the NDIS and when and how the NDIA will make decisions’ (Tune, 2019, p. 12), highlighting how the pivotal concept of VFM still requires further clarification within this large scheme.

Overall, the study sought to address the need for a better understanding of how the concepts of value, and VFM, are currently understood and operationalised by occupational therapists in home modification practice—with the aim of positively contributing to both policy and practice in this important area.

## 2 METHODS

### 2.1 Ethics

This study was approved by the Human Research Ethics Committee (HREC) at Griffith University under HREC Approval Number 2020/453.

### 2.2 Study design

This paper reports on the findings of a qualitative study, which formed the first phase of a multiphase project exploring the concepts of value and VFM in Australian home modification practice. In doing this, it sought answers to the question: ‘How are value and value for money currently understood and operationalised within home modification practice in Australia?’. Although the initial aim was to focus primarily on the concept of VFM, understanding value (and how it is operationalised) is critical to understandings of what constitutes a ‘valued outcome’ (Smith, 2009, p. 12). Constructivist grounded theory methodology (Charmaz, 2006, 2014) was selected for its potential to facilitate an inductive ‘ground up’ (Urquhart, 2013, p. 8) theory constructed directly from the experiences and understandings of occupational therapists at the frontlines of the home modification process.

Phase 1 of this project (reported on here) collected and analysed data from 20 occupational therapists experienced in home modification practice from across Australia. Phase 2 will bring together the perspectives of end users and other key stakeholders. As such, Phase 2 will facilitate comparison and contrast between the various perspectives to ‘triangulate’ (Neuman, 2011) and further contextualise the findings of Phase 1.

### 2.3 Research participants and recruitment

Participant recruitment was generated from both advertisements posted in professional online forums and through referral, with prospective participants registering expressions of interest via either online form or email. Twenty occupational therapists were recruited (from 27 expressions of interest), with the inclusion criteria that they currently worked as occupational therapists in Australia and had professional experience in home modifications (a distinction between simple and complex home modifications was not made). Experience ranged from under 1 year post‐graduate to over 25 years (mean 11.15 years). Participants resided in four different states in Australia (Queensland, New South Wales, Victoria, and Western Australia), and 14 had undertaken regional work at some point in their career.

The occupational therapists interviewed worked in government departments (hospital and community‐based), insurance agencies, and private practice (self‐employed and as employees)—with many having experience in multiple of these. All had experience working for at least two different funding bodies, with 28 different funders referenced. Of the 20 occupational therapists interviewed, 17 had conducted home modification work under the NDIS. The occupational therapists had experience with diverse cohorts—across a wide spectrum of age and disability type.

### 2.4 Data collection and analysis

Semi‐structured interviews were conducted in person, via telephone, and through virtual meeting platforms. Interviews ranged from 38 minutes to 2 hours 21 minutes duration (mean 63 minutes). All interviews were conducted by the lead researcher (first author), a non‐occupational therapist design professional who had knowledge and experience in home modifications. All interviews were recorded, transcribed, and de‐identified, for the purposes of analysis and coding. Appendix A outlines the main interview questions, however, consistent with a semi‐structured qualitative approach, participants also had scope to direct the conversation.

In line with constructivist grounded theory methods, participant recruitment, data collection, and constant comparative analysis were undertaken iteratively (Birks & Mills, 2011; Charmaz, 2006, 2014). Throughout the analytic process, interview transcripts were subjected to three stages of coding: initial manual coding (Charmaz, 2006, 2014), subsequent focussed, and advanced coding and sorting (Birks & Mills, 2011; Charmaz, 2014) within the qualitative research software NVivo v12. The use of NVivo also aided the testing of the emergent theory, enabling confirmation of the codes and categories that were most frequently emphasised in the data, and helped to organise and manage the rich data. As per constructivist grounded theory methods, emergent analytic themes (codes and categories) informed the ongoing recruitment, data collection, and analysis in a cyclical manner until theoretical ‘saturation’ (Glaser & Strauss, 1967) was reached. Throughout the process, the ‘crucial’ (Charmaz, 2014, p. 162) grounded theory method of memo‐writing was employed to aid category development and map the emergent conceptual relationships. This enabled the development and refinement of the grounded theory presented here.

## 3 FINDINGS

**FIGURE 1:**
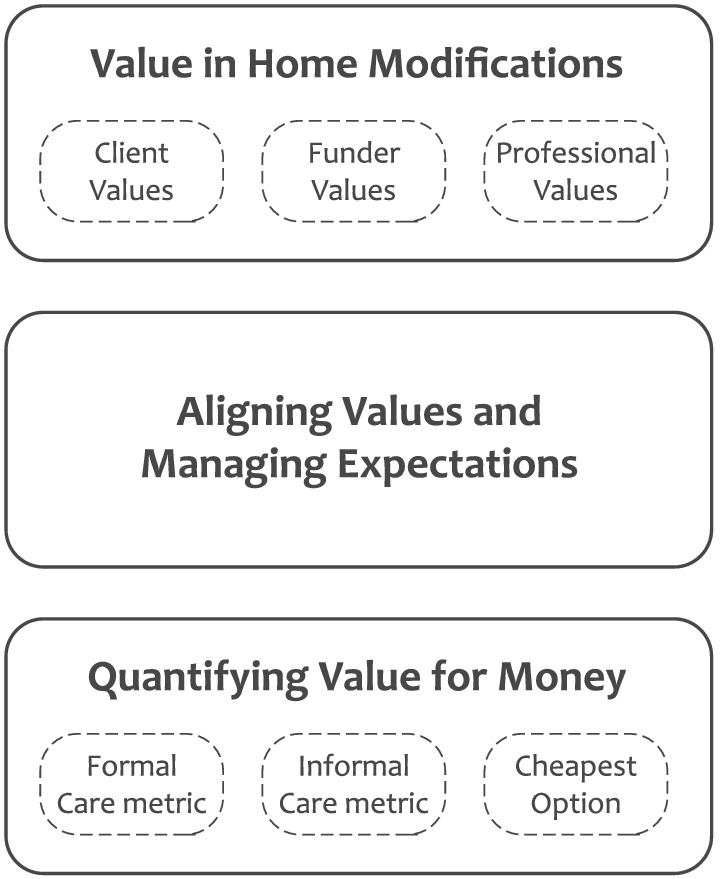
Overview of major themes and sub‐themes

In order to better understand VFM and ‘how resources are successfully transformed into valued outcomes’ (Smith, 2009, p. 12), it is necessary to understand what is valued. Figure 1 presents the high‐level themes and sub‐themes that emerged from the data, which are discussed in detail in the following sections.

The following section reports the analytic findings in relation to occupational therapists' perceptions of what different stakeholders valued. This is followed by the findings relating to VFM and the grounded theory derived from the study.

### 3.1 Understanding value in home modifications

#### 3.1.1 Understanding client values

Participating occupational therapists understood value in home modification as comprising both the client's and the funder's values, but they viewed these as being different. When describing clients' perceived values, strong emphasis was placed on clients valuing the aesthetic aspects of home modifications (34 references). This was referenced more frequently than any other client value, surpassing independence (31), quality of life (17), property value (10), control (8), dignity (7), mental health (7), and privacy (3). The theme of clients valuing aesthetics also overlapped with clients' concern about the property value of their homes ‘… inevitably, ultimately … someone will say well what's it going to look like, is it going to [de]value my house?’ (P03).

Examples were given of older clientele who had chosen to forgo necessary safety‐related modifications for fear that they would reduce the saleable value of the house and their children's inheritance. These discussions revealed tensions occupational therapists faced in reconciling form and function: ‘sometimes what people want is not necessarily what they need. So, sometimes it's kind of a compromise between meeting what they need functionally, and for safety, versus what they're wanting and often that's an aesthetic thing’ (P07). In the words of another: ‘the worst thing is aesthetics when it becomes an issue of aesthetics over function, I say … the [funder] aren't paying for home reno[vation] for you’ (P03). One solution proffered to this was requiring clients to self‐fund anything seen to be not purely functional: ‘If you need anything on top of it, that's all right, you can pay for it’ (P14). Other occupational therapists refused commissions altogether where they thought that the clients had: ‘high expectations aesthetically’ (P20) that could not be justified to a funder. It bears noting that the strong emphasis on the theme of aesthetics may, at least partly, be reflective of it being a value that occupational therapists frequently needed to negotiate with clients and connects with the theme of ‘managing expectations’ (discussed in detail later).

#### 3.1.2 Understanding funder values

The participating occupational therapists also understood the concept of value in home modification practice for funders to be at times distinctly different, and even in tension with, client values. As all the occupational therapists had experience working for at least two different funders, they were able to reflect on the differences that they had observed between schemes with evidence of value differences between, as well as within, schemes: ‘it does vary from body to body, and it also varies according to who is likely to be reviewing the application’ (P01). There was a general perception that funders were more likely to value proposals that resulted in cost savings elsewhere in their own scheme. One occupational therapist gave the example of a funder who was responsible for a greater portion of the general health‐care costs of those in the scheme (vs. other ‘disability specific’ funders) as being more amenable to funding modifications that could prevent health‐care expenditure such as hospitalisations from falls or injuries. Similarly, some funders responsible for lifetime care were referenced as being more open to proposals, which may cost more upfront, but over a lifetime proved cheaper than a series of smaller solutions: ‘[the funder] would prefer to do something once and do it really well … that hasn't always been the approach with the other schemes’ (P12). The occupational therapists also referenced certain funding bodies who had explicit rules around what constituted VFM in terms of longevity of use of the modification (i.e., that individuals should remain in the home for a minimum number of years post‐modification).

The occupational therapists perceived that some funders placed greater weight on some of their value criteria than others. For example, those who had worked under the NDIS (n = 17) perceived that VFM was the most heavily weighted criteria—as ascertained from feedback on applications. Some went so far as to say that they did not feel that the other values were considered at all: ‘the goals aren't even looked at, all the work that people have gone into writing their goals, they just seem to ignore that’ (P05). In the words of another occupational therapist, ‘… if NDIS is saying that they weigh all of those things equally, I'd strongly disagree with that statement and I would love to see some examples of when they think they've done that’ (P12).

#### 3.1.3 Professional values

Several occupational therapists spoke of their own professional values impacting their work, particularly regarding responsibilities for balancing clients' needs and values with costs to the funder. One occupational therapist gave the example of a client proposal they would not support because it emphasised aesthetics and ultimately increased the cost. This was echoed by another occupational therapist who relayed: ‘I'm a taxpayer, and everyone around me are taxpayers, and it's that money that is being spent’ (P08). Another emphasised the need to interrogate the underpinning societal values that inform decisions:

I guess it's a question for the whole society—do we want value for money to be the first value that drives our decision‐making? …. Do we as a society and as a community want to back people with disabilities and give them a shot at independence when it's not guaranteed? (P06)

Even those who claimed to always put their clients' goals and values ‘first and foremost’ (P3) acknowledged that they still did so within the parameters of what they deemed ‘reasonable …. what I would regard as reasonable, and there's probably some level of judgement in there which I bring to that’ (P3). Interestingly, whether an occupational therapist was employed directly by a funder or engaged directly by a client did not appear to alter how they viewed their professional role in managing values and expectations. This is addressed in more detail in the next theme.

### 3.2 Aligning values and managing expectations

As the previous sections have illustrated, the occupational therapists understood conceptions of value to vary between stakeholders. Consequently, they saw the aligning of client and funder values—particularly in relation to VFM—as a key part of their work. Close to two thirds of the occupational therapists spoke extensively about working to align values and manage expectations about what constituted value and VFM. Some described ‘tricky’ conversations (P16), working with clients who ‘struggle’ with the fact that ‘… what is going to work best for them may not represent the most VFM for the agency’ (P16). Clarity from funders about criteria or systems for determining value and VFM was identified by the occupational therapists as critical to successfully undertaking this work. The NDIS was cited frequently as an example of a scheme that did not provide sufficient clarity or transparency as to how value and VFM were determined (95 references from 16 participants), with inconsistent decision making also frequently raised as problematic (85 references from 15 participants) in that scheme. Some occupational therapists and organisations had stopped undertaking NDIS work altogether because their approval rates were so low that they felt their services could not offer VFM to clientele. There was some evidence that occupational therapists working directly with, and for, funders may have greater scope in responding to clients' values because they had more clarity around funding guidelines (and where flexibility may be afforded) as well as the ability to negotiate and exert influence through more direct access to decision makers. Many occupational therapists also cited schemes that worked in collaborative and team‐based ways as leading to ‘… better client outcomes …’ [P15], and ultimately better VFM as they saved both the occupational therapist’s, and the scheme’s staff time avoiding protracted negotiations and lengthy reports.

The ability to negotiate between client's and funder's values was seen to be a necessary skill. There were many references to persuasive methods such as ‘arguments’, ‘justifications’, and even ‘fighting’ by occupational therapists to get funders to see the value of a proposed modification. One occupational therapist described role playing a funder, requiring clients to ‘convince her’ by pretending they are speaking directly to the funder: ‘if you can convince me, I can convince the funding body. But if you can't convince me and I keep asking you questions, I can't convince anybody of that fact’ (P08). There were also reflections that without this experience ‘it's harder for the newbies who can sometimes get bullied into such situations and don't have the rebuttal skills to clients that are sometimes required for people's often outlandish expectations’ (P08).

### 3.3 Quantifying value for money

Interconnected with the value alignment work and managing expectations, many occupational therapists reported attempting to quantify VFM to persuade funders of the value of the modification. Three methods in particular were predominantly used to make a case for VFM: the use of formal care as a metric, informal care as a metric, and the cheapest option approach. These are discussed in more detail below.

#### 3.3.1 Formal care as a metric

Over half of the occupational therapists interviewed mentioned calculating the potential formal care savings that could be gained from a home modification. For many, the metric of paid caregiving represented a simple way of communicating VFM to funders and made otherwise less tangible valued outcomes, such as independence, more quantifiable. There were also references to potential paid care savings where the modifications might help a paid carer to undertake their role more efficiently. Some occupational therapists did reflect, however, that this kind of ‘economic rationalism’ (P05) could be problematic. For example, there were many physical conditions mentioned where increasing an individual's independence did not necessarily result in the need for less paid care. Individuals with deteriorating conditions were cited as cases where a home modification may aid in *retaining* current levels of function for longer but would not necessarily result in an immediate reduction of paid care: ‘occasionally, that might be the case, but nine times out of ten, most people are getting worse … or you're just trying to maintain their level of function’ (P05). There were also instances cited where clients had chosen to forgo a particular home modification because they were afraid that it would result in a reduction in their paid care entitlements: ‘the [funder] say if we give her that kitchen, we're reducing your funding in your plans to the value of the modification so you are signing your support worker hours [away]’ (P06).

#### 3.3.2 Informal care as a metric

Even more occupational therapists spoke of using informal care as a metric in VFM determinations. Because the cost of informal care can be ‘hard to quantify’ (P13), many spoke of calculating waged care substitution in the event that the informal caregiver was no longer able to perform the role due to a variety of reasons ranging from injury and stress through to relational breakdown. Some highlighted the wide range of impacts that were possible if the informal caregiver was no longer able to undertake this role:

if that family member is injured as a result of doing that task, then there will be not just showering tasks they can't do, there will be other supervision tasks, there will be meal preparation, there will be transporting tasks that will need to be funded so that the benefit of being able to sustain informal care is not just related to the task that is done in the area that we're trying to actually modify. (P01)

Others focussed more generally on ‘decreased carer burden’ (P19) and stress without necessarily quantifying it. These discussions also highlight the difficulties associated with trying to draw a line between the individual, their needs, and their immediate context. Occupational therapists spoke of assessing and accounting for the physical and mental health, and relational dynamics, of informal caregivers and other household members: ‘it's not like there's a one‐size‐fits‐all. It's very much a case by case, and how the individuals are coping, and the health needs of both people’ (P19). This was identified as important to understanding what the best solution might be, both currently and sustainably into the future.

One occupational therapist described a VFM calculation they made, which framed informal care as a reciprocal arrangement between the funder and the individual, who ‘… offers value for money to the [funder] by choosing to use informal supports rather than support workers …’ (P06). In the example given, the occupational therapist identified savings of $140,000 per year that the funder was making due to the individual's parents (who were in their 70s) electing to continue to care for their adult child. The occupational therapist used these ‘savings’ as part of the argument to request a $20,000 home modification, which was rejected by the funder.

#### 3.3.3 Cheapest option

In addition to the previous two approaches, the majority of occupational therapists also spoke about strategically working from ‘a low‐cost to high‐cost solution’ (P12), to identify the ‘cheapest way to get their goal met’ (P15). For many, this involved a ‘trial and error’ (P12) process where cheaper solutions were first trialled before larger interventions. There remained an awareness, however, that the cheapest price did not necessarily equate to the best outcome or VFM over time, as a series of smaller interventions may ultimately cost more than a single larger modification undertaken upfront. Some occupational therapists also spoke of demonstrating that the home modification was the cheapest option by comparing it with alternatives such as moving house (including stamp duty and relocation costs) or institutionalisation.

Of those who described using the cheapest option as their primary means of calculating VFM, there was some recognition that it created a tendency ‘to be not quite so exploratory and imaginative’ (P08) with their solutions.

In all of these approaches to calculating VFM, time was an important factor, with all but three of the occupational therapists speaking explicitly about considering changes to an individual's condition, needs, or context over time. In some cases, the trajectory of an individual's condition might be near impossible to accurately predict, and palliative cases were described as ‘one of the areas that is very challenging for working out value for money’ (P14). In one occupational therapist's words:

when I'm approaching funding bodies for some modifications for people who are palliative, I am keeping in mind that they will most likely only approve low‐cost modifications. So, when I'm speaking to those clients and the families, it's sort of sensitively putting it to them that the funding bodies won't approve expensive works. Sometimes there's a bit of pushback on that. ‘I've paid taxes all my life. I won't be helping with this.’ And trying to sensitively put it to them that it's government funding and it needs to benefit the majority of the population. And so, they will only do minor modifications at this stage, which is really hard to say to someone when they're in that situation. (P15)

**FIGURE 2:**
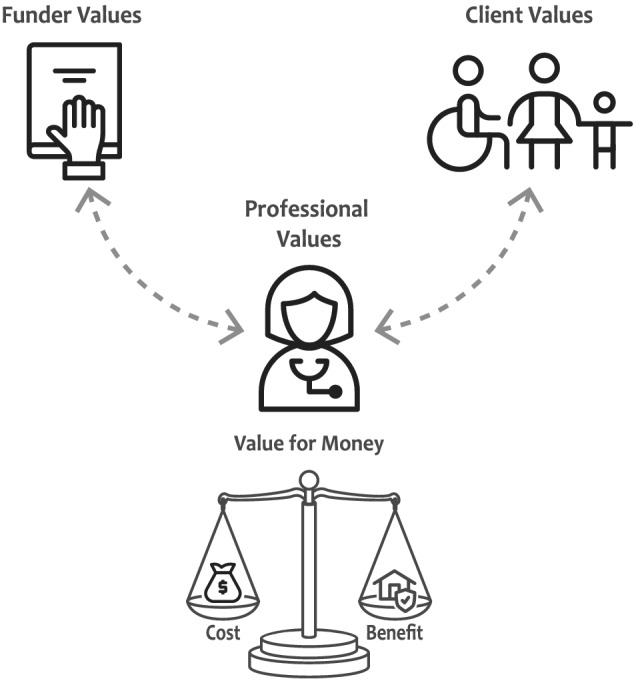
Value and value‐for‐money relational diagram

This quote also illustrates how larger issues of resource distribution and wider governmental and societal values were interwoven in these discussions. Figure 2 depicts the grounded theory that emerged from the study, and more specifically, it illustrates the intermediary position occupied by occupational therapists when attempting to align the client and funder values in order to achieve an outcome that is considered both valuable and VFM.

Regardless of whether occupational therapists were contracted by funders or by the end users, the work of aligning the different stakeholders' values to create outcomes that are seen by all as valuable, and as representing VFM, remained a central part of their home modification practice.

## 4 DISCUSSION

As this and other research has shown, VFM determinations are inextricably linked to understandings of value, which are ‘… multidimensional, and not necessarily quantifiable’ (Scharaschkin & McBride, 2016, p. 40). Consequently, VFM determinations are contingent upon the perspectives of the various stakeholders because ‘… they perceive costs and outcomes differently’ (Chiatti & Iwarsson, 2014, p. 325). The grounded theory that emerged from this research foregrounds the often unarticulated work that occupational therapists do in aligning disparate conceptualisations of value and VFM—often without external frameworks, metrics, or clear guidance as to how this should be done.

In framing the findings of this research, Lipsky's (2010) conceptualisation of occupational therapists as street‐level bureaucrats who both ‘effect and are affected by policy’ (Aldrich & Rudman, 2020, p. 137) is particularly useful. In addition to enacting policy, street‐level bureaucrats can also influence policy, which was evidenced within this study through frequent references to occupational therapists' ‘arguments’, ‘justifications’, and ‘fighting’ with funders. As frontline workers, occupational therapists are often required to deal with contradictions in order to make ‘judgements about how to act in a given situation because guiding policies speak to general rather than specific cases’ (Aldrich & Rudman, 2020, p. 139). This mirrors the ‘value alignment’ work that emerged from this study. Vanderkaay et al.'s (2020) study of occupational therapists' ethical decision‐making process has similarly observed occupational therapists engaging in a ‘push and pull process’ (p. 103) between stakeholders. Others have highlighted occupational therapists' unique positioning as both ‘gatekeepers’ and ‘advocates’ (Barbara & Curtin, 2008). Durocher et al. (2016) also spoke of occupational therapists navigating ‘ethical tensions related to balancing client priorities with those of health care services’ (p. 216). In each of these instances, occupational therapists are positioned at the intersection of not only their client's values and those of families, organisations, and systems but also their own personal values. In line with Lipsky's (2010) description of street‐level bureaucrats, occupational therapists are often required to embrace the contradiction of ‘values‐based human interaction on one hand and detached, organizationally‐driven service provision requirements on the other’ (Aldrich & Rudman, 2020, p. 138).

The grounded theory presented here also helps explain why the schemes with collaborative or team‐based approaches (i.e., those where the funder collaborates directly with professionals and clientele to co‐produce solutions) were reported more positively by occupational therapists. Specifically, this type of approach can facilitate more explicit communication of values and decision‐making criteria, enabling more direct ‘value alignment’ between stakeholders. They also can be seen to help span the—sometimes considerable—space between general policies and ‘specific cases’ (Aldrich & Rudman, 2020, p. 139). In cases where a funding scheme's size or structure does not easily support this type of collaborative approach, the need to supply occupational therapists with sufficient clarity around the values central to the funder and consistent frameworks, models, or tools for calculating VFM emerged as even more critical to achieving valued outcomes. Without these—significant resources can be wasted as occupational therapists work to elicit, communicate, translate, and align values between stakeholders.

The findings of this study have a range of practical and policy‐level implications for all stakeholders in the home modification process. For individuals engaging occupational therapy services, clear and direct conversations around their own values, and those of other stakeholders (such as funders), have the potential to produce faster and better quality home modification outcomes. From the funder perspective, there is an opportunity to realise significant resource savings through clearly communicated values and VFM criteria as a result of reduced negotiations, reviews, and administrative processes. Increased funder transparency is equally critical for occupational therapists particularly given the quantity and diversity of different funding schemes for home modifications in Australia (in this study, 28 different schemes were referenced by the 20 participants), each having their own values and VFM criteria. The occupational therapists' accounts of learning their ‘value alignment’ skills through years of on‐the‐job experience also signal an opportunity for educators to increase awareness and competency of students in this prior to graduation—particularly given the wider applicability of these skills within occupational therapy practice (outside of home modifications). There is also scope for future research to consider in greater depth how value and VFM determinations are made in cases where home modifications are self‐funded by clientele.

Looking beyond the direct stakeholders, this study has highlighted how VFM determinations are not simply economic calculations but rather involve complex considerations with ethical implications that span from the individual through to organisational and societal levels. As shown here, simple questions of ‘who pays?’ and ‘who benefits?’ give rise to larger and more complex social, political, professional, and economic questions of resource distribution and ethical decision making, which is particularly relevant in contexts characterised by limited resources. This research has also emphasised the need for further research, and the potential to develop professional tools, frameworks, and metrics that support those who undertake this important work.

## 5 LIMITATIONS

The occupational therapists' perspectives represented here can be seen to be limited by the relatively small sample size. Further, as the sample did not include participants from every state and territory in Australia, the findings are not expected to be representative of the full diversity of experiences across the different contexts and regions. As the project phase presented here drew solely on the accounts provided by occupational therapists—references to other stakeholders' actions or motivations need to be considered accordingly. Future phases of this research—which approach the subject matter from a wider range of stakeholder perspectives—have been purposefully designed to address this and allow the subject area to be better understood from different viewpoints. Within this study, the range of client values discussed in the interviews were also not presented in‐depth here but form a more central focus of Phase 2 of the larger project. This increased focus on client values will also help ensure that findings are triangulated and that the grounded theory developed is tested and continues to evolve.

## CONFLICTS OF INTEREST

The authors do not have any actual or perceived conflicts of interest to declare.

## AUTHOR CONTRIBUTIONS

The authors declare that this is original work and all authors met authorship criteria. G. B. and M. F. were involved in the study conceptualisation and design. G. B. collected and analysed the data and drafted the manuscript. M. F. contributed to the ongoing analytic development of the project and manuscript reviewing and editing at multiple key points. C. G. contributed to the literature review and manuscript editing. The final manuscript was reviewed and approved by all authors prior to submission.

## Data Availability

The data that support the findings of this study are available from the corresponding author upon reasonable request.

## APPENDIX A SCHEDULE OF INTERVIEW QUESTIONS

**Table:**

Primary interview question	Sub‐questions and prompts
Are you able to give some background on your work in home modifications?	How long have you worked in home modifications? Which funding bodies have you worked for? Do you currently, or have you ever, worked in regional or remote areas? (*If yes*) Are there any specific challenges or opportunities that you have noticed in relation to working regionally?
*This study is looking at the concepts of* value *and* value for money *in home modification practice. I would like to break our chat into two parts, the first part looking at* value for money *and the second more closely at* value.	
Looking first at the concept of value for money in home modifications—which is not always a simple thing to define—are you able to share how you personally define value for money in your work?	How have you demonstrated, or quantified, value for money to funders? Are there any particular metrics you use to quantify value for money to funders? (*If yes*) Please describe.
Have you ever noticed a difference between how you define value for money and how a funding body has defined it?	(*If yes*) Can you provide examples of instances when your definition of value for money and a funding body's definition have differed? Do you feel there is enough clarity/guidance provided by funders around what constitutes value for money? Have you observed differences between funding bodies as to what is considered value for money? Have you observed any differences in determinations of value for money with the same funding body? Have you noticed any changes in the way that funders define value for money over time?
*If participant has worked with the NDIS ‐* Section 34 of the NDIA Scheme Act requires that ‘reasonable and necessary supports’ meet six criteria (*interviewer to read these out if necessary*).	
In your experience (including feedback you have received on applications) do you feel that all of these criteria are weighted evenly?	(*If no*) Which do you perceive to be most strongly emphasised? (*If no*) Which do you perceived to be least emphasised?
Now moving from *value for money* to the concept of *value*. Home modifications can be seen as providing value to individuals and funders in a number of ways.	
Do you see any differences between a modification being ‘valuable’ and representing ‘value for money’?	(*If yes*) What do you see as the main differences?
Are there times when you have felt that a modification would be valuable for a client (or their family) but you don't think it represents value for money for the funder?	Do you have any specific examples that come to mind?
Is there anything that could be done to make it easier for OT's/assessors to decide what represents value and value for money?	*If clarification is required*: For example, clearer guidelines, tools or agreed metrics?
Is there anything that we haven't covered that you would like to add, or think is important to draw attention to?	
